# Strengthening the Healthcare System in Low- and Middle-income Countries by Integrating Emergency Care Capacities

**DOI:** 10.31662/jmaj.2018-0041

**Published:** 2019-06-28

**Authors:** Shinji Nakahara, Masao Ichikawa, Tetsuya Sakamoto

**Affiliations:** 1 Department of Emergency Medicine, Teikyo University School of Medicine, Tokyo, Japan; 2 Faculty of Medicine, University of Tsukuba, Tsukuba, Japan

**Keywords:** Primary healthcare, emergency care, strengthening healthcare system, low- and middle-income countries

## Abstract

Primary healthcare (PHC) principles provide a framework for strengthening the healthcare system to tackle increasing and diversifying health needs in low- and middle-income countries (LMICs). Currently, PHC systems in LMICs require expanded care capabilities in order to deal with noncommunicable diseases and injuries, including emergency conditions. In this article, we discuss the possibility of applying PHC principles to emergency care in LMICs and integrating emergency care into PHC; such principles include providing first points of contact with healthcare through nonprofessional providers close to communities in order to improve accessibility, providing high-quality (i.e., comprehensive, coordinated, and continuous) primary care, and addressing primary causes of ill-health through community empowerment. These principles are applicable to emergency care, which has the same attributes: it also requires increasing first points of contact through layperson first aid and the ambulance system, and it also provides comprehensive care for diverse diseases and injuries, with various facilities and personnel involved in its coordinated and continuous delivery; collective community actions also develop and strengthen the emergency care system, particularly through components outside the health sector (e.g., transport, communication, and mutual aid). Integrating emergency care into PHC could enhance the general health system and is more efficient than having separate systems.

## Introduction

Recent epidemiological transitions in low- and middle-income countries (LMICs), along with the increase of diseases that potentially require emergency care, have required shifts in various healthcare capacities ^(1), (2), (3), (4)^. While infectious diseases are gradually being brought under control, noncommunicable diseases, such as hypertension and diabetes, which may require acute care with severe symptoms (e.g., heart attack or stroke), are increasing. Road traffic injuries are also increasing owing to rapid motorization. In addition, the possibility remains of maternal conditions and infectious diseases becoming critical.

In order to respond to the increasingly diversifying healthcare demand, integrating emergency care would strengthen the entire healthcare system and would be more cost-effective than operating separate systems ^(5), (6)^. The primary healthcare (PHC) principles that have provided a basic framework for strengthening the already existing healthcare system and helped achieve health equity in LMICs can also be applied to the development of emergency care capabilities in general healthcare systems. These principles assist in the response to diversifying health needs and address health inequities. In addition, there are commonalities between PHC and emergency care as both involve laypeople (i.e., nonprofessional providers) as the first point of contact with the care and address a broad range of health problems ^(7), (8), (9)^.

PHC principles originated in the Alma-Ata Declaration of 1978 and are based on the failures of facility-oriented healthcare programs and the successes of community-based healthcare programs ^(10)^. Investment in tertiary care hospitals in urban areas was the predominant approach in newly independent countries before the Alma-Ata Declaration, but these investments did not improve access to health services or the health of the population, particularly in rural areas. In contrast, some successful community-based programs, in which basic health services were provided by nonprofessional personnel, such as community health workers (CHWs) or “barefoot doctors,” inspired the PHC principles ^(11), (12)^.

Likewise, high-income country (HIC) models of emergency care, which depend on an ambulance system with professional paramedics or physicians and emergency departments in tertiary care hospitals, do not adequately reach rural or underserved populations in LMICs ^(6), (13), (14)^. Instead, the use of community-based models with laypersons or nonprofessional providers can improve access to care and strengthen capacities in rural communities ^(7), (9), (13)^. In turn, emergency care skills, including the prompt arrangement of referrals, transportation, financing, and communication with other providers, when integrated into the community-based PHC system, can improve the quality of general health services.

In this article, we discuss the extent to which PHC principles are applicable to the development of community-based emergency care, focusing on the health needs in LMICs, particularly in resource-constrained remote settings. Our discussion followed the three different meanings of “primary” in the key PHC principles, which are bringing the primary (first) points of contact with the healthcare system closer to the community, providing high-quality primary care, and addressing the primary (root) causes of ill-health ^(7), (15), (16)^. For each of these three components, we examined the definition by reviewing the existing literature and identified similarities between PHC and emergency care in terms of already existing problems and potential solutions. We then examined whether the integration of emergency care into PHC would strengthen the general healthcare system by enhancing the care quality and community participation.

## First Contact with Healthcare

The basic premise of PHC is that the healthcare system should bring care as close as possible to individuals by placing the first points of contact with the healthcare system in communities ^(9), (10)^. Increasing the points of contact with healthcare is expected to fill in the gaps in access to healthcare. The roles performed by the first points of contact include visiting communities to identify health problems and dealing with those who visit health facilities ^(17)^. In countries where there are insufficient professional medical personnel and facilities, these tasks have been shifted from medical professionals in hospitals to lay providers (e.g., CHWs) ^(18), (19), (20)^. CHWs are based in village health centers and provide basic and preventive healthcare using simple techniques appropriate for nonprofessionals. Although rigorous evaluation studies are lacking, CHW programs have the potential to increase access to and utilization of health services and to improve health outcomes ^(18), (19), (21)^.

Emergency medical services (EMSs), also referred to as ambulance services, including first aid provided by laypersons, are another strategy to bring points of contact with healthcare services to the community. Nonphysician and physician ambulance personnel provide care before reaching the hospital depending on their skill level. The first aid provided by laypersons is an important component of emergency care even in HICs, where they are an indispensable part of the chain of survival ^(22), (23)^. EMSs also provide transport services, which can help address inequitable access to care. Without such services, severely ill or injured patients living far from healthcare facilities, particularly those who are poor, would face significant barriers to access services, causing a delay in treatment ^(24)^.

Integrating PHC with emergency care may strengthen the healthcare system as a whole, given that both systems require lay providers as the first points of contact in communities. Having points of contact provide both emergency and nonemergency care is more efficient than having separate service systems ^(5), (6)^. Health center staffs and CHWs provide emergency care; in turn, emergency care providers may have the opportunity to contribute to general health services. In an example from HICs, paramedic practitioners make home visits to treat minor diseases, perform assessments of patients’ daily lives and social aspects, and communicate with family physicians ^(25)^.

However, HIC models of emergency care systems consisting of hospital-based specialized care and formal EMS systems are too expensive for resource-constrained countries to provide adequate coverage to remote and dispersedly populated areas ^(6), (13), (14)^. This situation evokes the failure of facility-based medical models in the pre-Alma-Ata era. Investing in HIC models, despite being seemingly attractive to external donors seeking rapid outcomes, would concentrate limited resources in urban areas, only widening the gap ^(6), (10), (14), (26), (27), (28)^.

Instead, community-based care provided by nonprofessional personnel should be emphasized. Similar to the pre-Alma-Ata era, there have been successful community-based emergency care projects that have trained nonprofessional personnel to reach rural communities using appropriate technologies. Trauma first aid (e.g., hemorrhage control) administered by laypersons is an example of a promising project ^(29)^. In Ghana and Madagascar, taxi drivers who are likely to witness traffic injuries were trained to provide trauma first aid ^(30), (31)^; in Uganda and South Africa, community residents, police, and commercial drivers were also trained in trauma first aid ^(13), (14), (32)^. A successful example of integrating PHC with emergency care has been reported in Iraq and Cambodia: emergency care training of CHWs (PHC personnel) and lay first responders improved the survival of patients with trauma ^(33)^. This example was expanded by including nurses and physicians of Iraqi hospitals and was replicated in Iran ^(34), (35), (36)^. Such informal programs may come to be accepted as part of a formal healthcare system, as was the case with the introduction of PHC ^(37)^.

## Primary Care

Although PHC is not a version of primary care, high-quality primary care should be part of PHC in order to meet the various health needs of the whole population ^(7)^. Starfield ^(9), (15), (38)^ defined “primary care” as first-contact, continuous (long-term, person-centered), comprehensive, coordinated care. This concept was envisaged in the Alma-Ata Declaration ^(10)^. In this section, we will discuss how integrating emergency services with primary care would enhance these aspects of high-quality primary care, except for first-contact care, which we have already discussed. As emergency care has a comprehensive nature and involves initial assessment and subsequent referral coordination, this would strengthen primary care management and result in increased user satisfaction and more effective use of services ^(5), (6), (39), (40), (41), (42)^.

### Comprehensiveness

Primary care should provide a comprehensive set of health services to respond to the increasing expectations in healthcare and to diversifying health problems due to epidemiological transition in LMICs (e.g., increasing lifestyle-related chronic diseases, injuries, and health problems due to climate change and disasters) ^(9), (15)^. Primary care should be the entry point to all types of services, whether those services are directly available at the point or whether users are connected to other providers as needed, which is key to user satisfaction. Selective approaches that provide limited services used to be the mainstay programs, as they were preferred by governments and donor agencies for their rapid results and easily measurable outcomes from short-term inputs ^(7), (9), (27)^. However, comprehensive approaches are more effective in addressing health issues than selective approaches are ^(43)^.

Emergency medical care also has a comprehensive nature: it targets patients with acute conditions regardless of the type of disease, as any disease can become critical. Therefore, selective approaches do not fit emergency care systems well. When selective emergency service programs were implemented, they naturally expanded their target population. For example, programs providing transport for obstetric emergency cases focused on pregnant women but were also utilized by men and children with severe illnesses and injuries ^(44), (45)^. Rather than having separate emergency care systems for different diseases, it is more efficient to have a single system that provides services for all diseases ^(46)^.

Integrating emergency care into primary care would contribute to enhancing the comprehensiveness of primary care ^(5), (6), (41), (42)^. Emergency care abilities in stabilizing, diagnosing, and triaging acute conditions would strengthen primary care, enabling it to deal with various healthcare needs. Instead of disease type, primary care personnel should be able to differentiate between emergency and nonemergency conditions and should have different protocols depending on the time sensitivity of conditions ^(7), (40)^.

### Coordination

When in need of specialized care, patients should be referred from primary care to secondary or tertiary care depending on their needs, with appropriate timing. This requires that primary care providers be aware of where and how to obtain the necessary resources for patients, be integrated in the healthcare network, and be able to arrange referral procedures and communicate with other providers ^(7), (9)^. With the current information technology, primary care can be easily connected with specialists (e.g., through cell phones or social network services) to obtain specialist advice or send the patient’s information before the referral ^(7)^. Good coordination abilities would also improve the gatekeeper function of primary care. User satisfaction with and trust in the coordination abilities of primary care staff would reduce the rate of self-referrals ^(39), (40)^.

Emergency referrals require a wider range of resources and stronger coordination or negotiation abilities, such as responsiveness (available 24 h), appropriate decision-making regarding who should be referred and when, means and skills to communicate with other services, and transport and financing arrangements (risk sharing or fee exemption scheme). Integrating emergency care into primary care would streamline the overall referral process in primary care and district health systems. In fact, in areas with no formal ambulance system, primary care personnel are expected to coordinate referrals of emergency as well as nonemergency cases. For example, a study in Cambodia showed that the majority of patients with emergency conditions sought help at the nearest health centers ^(40)^.

Transport arrangements, whether in primary care facilities or the community, are particularly critical for emergency transfers and referrals in areas where there is no formal EMS system ^(40)^. Successful examples in PHC settings include the following: motorcycle ambulances ^(45)^, tuk-tuk vehicles stationed in health centers ^(40)^, commercial driver mobilization^(31), (44), (47)^, and solar-powered radio network and rudimentary vehicle stationed in a district hospital ^(48)^.

Addressing financial barriers would facilitate the utilization of emergency services via primary care ^(40)^. Primary care should be able to directly manage risk sharing or cost exemption schemes or promptly contact community-based schemes. In addition to health service costs, long-distance transport costs and opportunity costs (e.g., loss of income for a long time) are so high in emergency cases that patients and their families may go bankrupt ^(24), (39), (49)^. Such costs can be an immediate barrier to referrals that a risk-sharing system should preferably cover ^(24), (47), (50), (51), (52), (53), (54)^.

### Continuity

The meaning of care continuity is twofold: temporal and interprovider continuity ^(9), (24), (38)^. In either case, patient information should be maintained and shared by all service providers. Temporal continuity refers to treating the patient with a consistent approach over time according to an understanding of the patient and his/her circumstances, usually by a regular provider in primary care ^(9)^. Interprovider continuity is required when patients need various types of specialized care and when all specialists involved in their care should take a consistent approach.

Emergency referrals require interprovider communication to share patient information promptly and accurately; the integration of emergency care would strengthen this aspect of primary care. Referral communication functions in various ways ^(24)^. In upward referral of emergency cases, primary care providers send information on the patients’ history and conditions or may request specialists’ advice regarding first aid and referral necessity. In downward referral of emergency cases, patients return from tertiary to primary care after receiving definitive acute care, with information on the acute treatment received and necessity of rehabilitation and aftercare. Some patients may require welfare services to return to the community. In practice, interprovider communication, key to information sharing, has room for improvement. For instance, information is not appropriately conveyed via referral letters or telephone when patients are referred ^(24), (55), (56)^. However, new information technologies have enabled easier interprovider communication. For example, by utilizing social networking services, images can be shared with no effort and expert advice can be easily obtained.

## Primary Causes of Ill-health

Addressing the primary (root) causes of ill-health to achieve health equity is the core value of PHC and differentiates it from “primary care.” Health equity is an aspect of social justice, given that everyone has the right to attain the highest possible level of health ^(8), (16), (57)^. Health status is determined not only by biological factors but also by social factors, such as poverty, personal behaviors, lifestyle, interaction with family members and neighbors, support from the community, access to social and health services, living environments, and social and cultural environments ^(9), (58)^. The social determinants of health (SDHs) are usually unevenly distributed in the society and are potentially modifiable ^(58)^. Modifying SDHs that are outside the health sector is a component of PHC actions, which requires sector-wide public policies and input from outside the health sector. In contrast, primary care focuses on health service delivery and can be fulfilled within the health sector.

SDHs also influence the incidence of emergency conditions (e.g., trauma and stroke), access to emergency care, and outcomes, although most of the evidence comes from HICs. Access to healthcare is also an SDH, as poor access to care directly affects the prognosis of severely ill and injured patients. For example, ethnic disparities in stroke incidence and access to care are prevalent and result from modifiable social factors, although genetic factors also contribute to the differences in incidence ^(59)^. Similarly, socioeconomic status, ethnicity, and gender are associated with access to care and outcomes of myocardial infarction ^(60)^. Low socioeconomic status is also associated with a higher risk of trauma ^(61), (62)^. Studies in LMICs also show an association of socioeconomic status with risk behaviors and incidence of myocardial infarction ^(63), (64)^.

Therefore, public policies, as well as public health policies, should aim to address the uneven distribution of SDHs including healthcare resources ^(8), (10), (65)^. The most underserved populations should be prioritized to minimize the inequalities, because starting from the “easiest to reach” populations, who already have good access to services, would simply widen the gap ^(16), (66)^. This approach is relevant to both primary and emergency care, and their integration would be promising in minimizing the gap ^(6), (33)^. For example, investment in lay providers increases service access points in underserved areas.

In addition to increasing the first points of contact to healthcare, strengthening district- or provincial-level health systems should also put a special emphasis on remote areas to address uneven access to health services. District or provincial hospitals need to have capabilities to provide appropriate definitive or specialized care to severely ill and injured patients coming to the hospitals referred from primary care. Otherwise, patients referred to a district or provincial hospital find that the care that they require is not available there and must travel to a national hospital in the capital city, causing further delay ^(1), (67), (68)^.

Community-level leadership, termed “community participation” in the Alma-Ata Declaration, is crucial for dealing with local health issues and uneven distribution of SDHs ^(69)^. Community participation is a key feature of PHC but has been defined in various ways. The fundamental factor is that community residents contribute to delivering health services as part of the service system and are not simply its beneficiaries ^(7), (69), (70)^. More importantly, their active participation and decision-making power would foster collective action by the community that contributes to change (i.e., community solidarity) ^(8), (70)^. In turn, this community action will help individuals act with confidence to improve their lives and environments ^(70)^.

Community-level collective actions are even more critical when providing emergency care in resource-constrained settings in remote areas that HIC models of ambulance systems cannot reach. Community residents should play a central role and serve as first responders and managers of the various systems outside the health services, such as transportation, communication, and financial protection ^(24)^. Fortunately, it is easy to set clear targets regarding these elements (e.g., vehicle arrangement and pooling money) so that residents can plan and manage them. There have been successful innovative attempts in communities to mobilize laypersons as first responders or to establish mutual aid systems to arrange transportation and money for emergencies ^(33), (44), (45), (47), (48), (50), (71), (72)^.

Such community-level actions show commonalities that are favorable to the integration of emergency care with PHC, although organization structures may differ from community to community. The above-mentioned examples are a simple expansion of already existing PHC programs (training of PHC personnel) ^(33)^ or inclusive by nature (mutual aid or transport arrangements that can cover any health problems) ^(44), (45), (47), (48), (50), (71), (72)^. If various providers are involved in different actions (e.g., commercial drivers in transport arrangements, village leaders in mutual aid programs, and village health volunteers in first-responder programs), they are coordinated under the community leadership.

Attempts to develop community-level collective actions to improve the healthcare system, in turn, may enhance community empowerment and solidarity ^(73)^. For example, training of lay community volunteers in trauma first aid has been found to change their perceptions of their abilities ^(33), (73)^. Before the training, they did not know how to help severely injured patients, feared doing something wrong, and often did nothing; after the training, they had the confidence to help injured patients. Establishing mutual aid or transport arrangement systems may also foster community stewardship of such systems.

## Further Actions

Here, we examined the similarities between PHC and emergency care through a literature review and explored the possibility of integrating these services to strengthen the general healthcare system. Nonprofessional community healthcare providers, such as CHWs and health volunteers (primary points of contact for PHC), who provide preventive care and simple curative care, can also serve as the first points of contact for emergency care, if they have first-aid training and the ability to communicate with the EMS or hospitals. Primary care providers can improve their ability to provide care by learning emergency care skills and streamlining their referral network, which would increase user satisfaction with their services. Community-level collective actions, such as mutual aid and transport arrangements, can contribute to minimizing inequitable access to healthcare (primary cause), which would lead to community empowerment and improved general healthcare.

Meanwhile, integrating enhanced emergency care into the general healthcare systems requires strong national-level leadership with a broader vision to organize actions that can integrate stakeholders external to the health sector ^(9)^. First, nationwide training should be conducted hierarchically by restructuring the training systems. For example, CHWs or primary-care-level personnel should train lay personnel (village health volunteers), district hospital personnel should train CHWs and primary care personnel, and provincial or national hospital personnel should train district-level personnel ^(33), (34), (35), (36)^. Second, many factors of emergency care, particularly prehospital care, are beyond the control of the health sector (e.g., transport, communication, and public safety). Prevention strategies aimed at addressing the uneven distribution of SDHs also require input from outside the health sector. However, health policymakers may have concerns and hesitance about dealing with political and social issues that are beyond the realm of the health sector ^(65), (74)^. Superordinate government bodies that supervise and control related sectors may be needed.

Local activities can only be exercised or activated within legal or institutional frameworks; national-level plans could offer a guide, and safeguard systems may be needed to protect those who participate in the activities ^(75)^. For example, community initiatives to develop a lay first-responder system cannot work without legislation that enables laypersons to provide emergency care and a protection mechanism in the case of adverse events (both to beneficiaries and to providers).

More research and evaluation activities based on high-quality data need to be conducted, as the health system should be improved according to evidence-based evaluations and recommendations. Currently, there is only weak evidence supporting the integration of community-based emergency and primary care, or even the PHC programs targeting maternal and child health, infectious diseases, and nutritional conditions ^(18), (19), (21)^. Identifying and examining the distribution of risk factors of ill-health, including emergency conditions, is an important research agenda contributing to public health strategies to prevent ill-health and decrease health inequities in LMICs ^(59), (64)^.

## Conclusions

Lessons learned from PHC experiences can be applied to the development of emergency care in LMICs; in turn, the capabilities required in emergency care may enhance the whole healthcare system. Epidemiological transitions in LMICs require care capacities in the healthcare system to deal with noncommunicable diseases and injuries, which should include emergency care abilities. It may be more efficient to have the same points of contact with healthcare for emergency and nonemergency cases than to have separate systems, and this can be achieved by providing emergency care training to primary care providers. This can also enhance the comprehensiveness of care and coordination abilities. The emergency care system requires components outside the health service, such as transportation and mutual aid systems that have clear targets. Entitling community residents to develop community-based management schemes would foster stewardship and empowerment.

## Article Information

### Conflicts of Interest

None

### Sources of Funding

This work was supported by JSPS KAKENHI grant number [16K11422].

### Author Contributions

All authors contributed to the concept of the manuscript, interpretation of the literature, and critical review of the manuscript and approved the final version. SN reviewed the literature and drafted the manuscript.

### Approval by Institutional Review Board (IRB)

Not applicable for review articles.
